# Atrial fibrillation in cryptogenic stroke and TIA patients in The Nordic Atrial Fibrillation and Stroke (NOR-FIB) Study: Main results

**DOI:** 10.1177/23969873221123122

**Published:** 2022-10-20

**Authors:** B Ratajczak-Tretel, A Tancin Lambert, R Al-Ani, K Arntzen, GK Bakkejord, HMO Bekkeseth, V Bjerkeli, G Eldøen, A Gulsvik, B Halvorsen, GA Høie, H Ihle-Hansen, H Ihle-Hansen, S Ingebrigtsen, H Johansen, C Kremer, SB Krogseth, C Kruuse, M Kurz, I Nakstad, V Novotny, H Næss, R Qazi, MK Rezaj, DM Rørholt, LH Steffensen, J Sømark, H Tobro, TC Truelsen, L Wassvik, KL Ægidius, D Atar, AH Aamodt

**Affiliations:** 1 Department of Neurology, Østfold Hospital Trust, Grålum, Norway; 2 Institute of Clinical Medicine, University of Oslo, Oslo, Norway; 3 Department of Cardiology, Østfold Hospital Trust, Grålum, Norway; 4 Department for Neurology, Nordlandssykehuset, Bodø, Norway; 5 Department of Neurology, Lillehammer Hospital, Innlandet Hospital Trust, Lillehammer, Norway; 6 Research Institute of Internal Medicine, Oslo University Hospital, Oslo, Norway; 7 Department of Neurology, Molde Hospital, Molde, Norway; 8 Department of Internal Medicine, Diakonhjemmet Hospital, Oslo, Norway; 9 Ullevål, Stroke Unit, Department of Neurology, Oslo University Hospital, Oslo, Norway; 10 Department of Internal Medicine, Vestre Viken Hospital Trust, Bærum Hospital, Gjettum, Norway; 11 Department of Neurology, University Hospital of North Norway, Tromsø, Norway; 12 Department of Neurology, Oslo University Hospital, Rikshospitalet, Oslo, Norway; 13 Department of Neurology, Department of Clinical Sciences Lund University, Skåne University Hospital, Malmö, Sweden; 14 Department of Neurology, Vestfold Hospital, Tønsberg, Norway; 15 Department of Neurology, Herlev Gentofte Hospital, Herlev, Denmark; 16 Department of Neurology, Stavanger University Hospital, Stavanger, Norway; 17 Department of Neurology, Vestre Viken Hospital Trust, Drammen Hospital, Drammen, Norway; 18 Department of Neurology, Haukeland University Hospital, Bergen, Norway; 19 Department of Neurology, Telemark Hospital, Skien, Norway; 20 Department of Neurology, Rigshospitalet University Hospital, Copenhagen, Denmark; 21 Department of Neurology, Bispebjerg University Hospital, Copenhagen, Denmark; 22 Department of Cardiology, Oslo University Hospital, Ullevål, Oslo, Norway; 23 Department of neuromedicine and movement science, the Norwegian University of Science and Technology, Trondheim, Norway

**Keywords:** Cryptogenic stroke, atrial fibrillation, insertable cardiac monitor, biomarkers, arrhythmia monitoring, anticoagulation, secondary prevention

## Abstract

**Introduction:**

Secondary stroke prevention depends on proper identification of the underlying etiology and initiation of optimal treatment after the index event. The aim of the NOR-FIB study was to detect and quantify underlying atrial fibrillation (AF) in patients with cryptogenic stroke (CS) or transient ischaemic attack (TIA) using insertable cardiac monitor (ICM), to optimise secondary prevention, and to test the feasibility of ICM usage for stroke physicians.

**Patients and methods:**

Prospective observational international multicenter real-life study of CS and TIA patients monitored for 12 months with ICM (Reveal LINQ) for AF detection.

**Results:**

ICM insertion was performed in 91.5% by stroke physicians, within median 9 days after index event. Paroxysmal AF was diagnosed in 74 out of 259 patients (28.6%), detected early after ICM insertion (mean 48 ± 52 days) in 86.5% of patients. AF patients were older (72.6 vs 62.2; *p* < 0.001), had higher pre-stroke CHA₂DS₂-VASc score (median 3 vs 2; *p* < 0.001) and admission NIHSS (median 2 vs 1; *p* = 0.001); and more often hypertension (*p* = 0.045) and dyslipidaemia (*p* = 0.005) than non-AF patients. The arrhythmia was recurrent in 91.9% and asymptomatic in 93.2%. At 12-month follow-up anticoagulants usage was 97.3%.

**Discussion and conclusions:**

ICM was an effective tool for diagnosing underlying AF, capturing AF in 29% of the CS and TIA patients. AF was asymptomatic in most cases and would mainly have gone undiagnosed without ICM. The insertion and use of ICM was feasible for stroke physicians in stroke units.

## Graphical abstract

**Graphical abstract:**
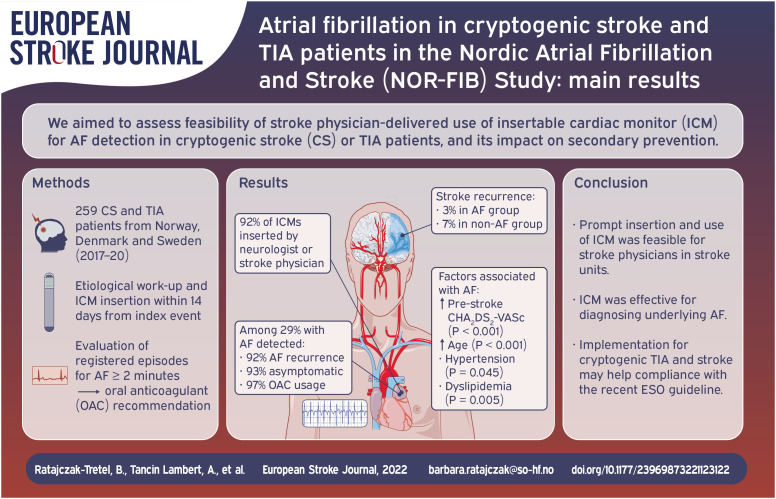

## Introduction

Ischaemic stroke without identifiable cause, where the etiology is undetermined after extensive investigation, is defined as cryptogenic (CS).^1^ Approximately one in four ischaemic strokes remain cryptogenic.^2^ Cardioembolism due to occult atrial fibrillation (AF) seems to be one of the most common causes of CS identified by thorough investigations. Current standard recommendation for secondary prevention with antiplatelet drugs may therefore not be sufficient for such heterogeneous group of patients. However, empiric treatment with oral anticoagulants (OAC) among embolic stroke of undetermined source (ESUS) patients without verified AF is not recommended.^3^ Recurrent strokes caused by AF may only be prevented if more patients undergo appropriate cardiac rhythm evaluation. Prolonged monitoring for about 30 days has been recommended since 2014 (Class IIA LoE C) and continuous ECG monitoring for at least 72 h whenever possible since 2020.^4,5^ Furthermore, several trial results have provided evidence in favour of longer continuous ECG monitoring and especially supported the use of insertable cardiac monitors (ICMs) in evaluating patients with CS.^6–8^ Recent meta-analyses show ICMs as more effective modality than conventional strategies revealing AF in 16%–34% of the CS patients.^9,10^

Notwithstanding, ICMs have so far not been routinely offered to eligible patients, mostly due to high costs, and until recently were not implemented as a preferred tool in any stroke guidelines.^3^ Strategies contributing to the proper use of resources and reducing evaluation costs may improve that. Identifying clinical algorithms and biomarkers to select CS patients with the highest possibility of detecting AF by subsequent prolonged monitoring may be a useful tool.

The main purpose of the Nordic Atrial Fibrillation and Stroke (NOR-FIB) study was to detect and quantify AF burden in patients with CS or cryptogenic TIA under continuous 12 months cardiac rhythm monitoring with an ICM managed by stroke physicians. At the time the study was initiated and conducted, ICMs were mostly managed by cardiologists and unavailable for stroke patients in the Nordic countries being occasionally used in CS patients. We sought therefore to test the feasibility of early ICM insertion by stroke physicians and long-term cardiac rhythm monitoring organised by the stroke units to optimise secondary prevention. This article presents the main results of the NOR-FIB study regarding arrhythmia detection and ICM usage by stroke physicians.

## Patients and methods

### Study design

**Figure 1.**
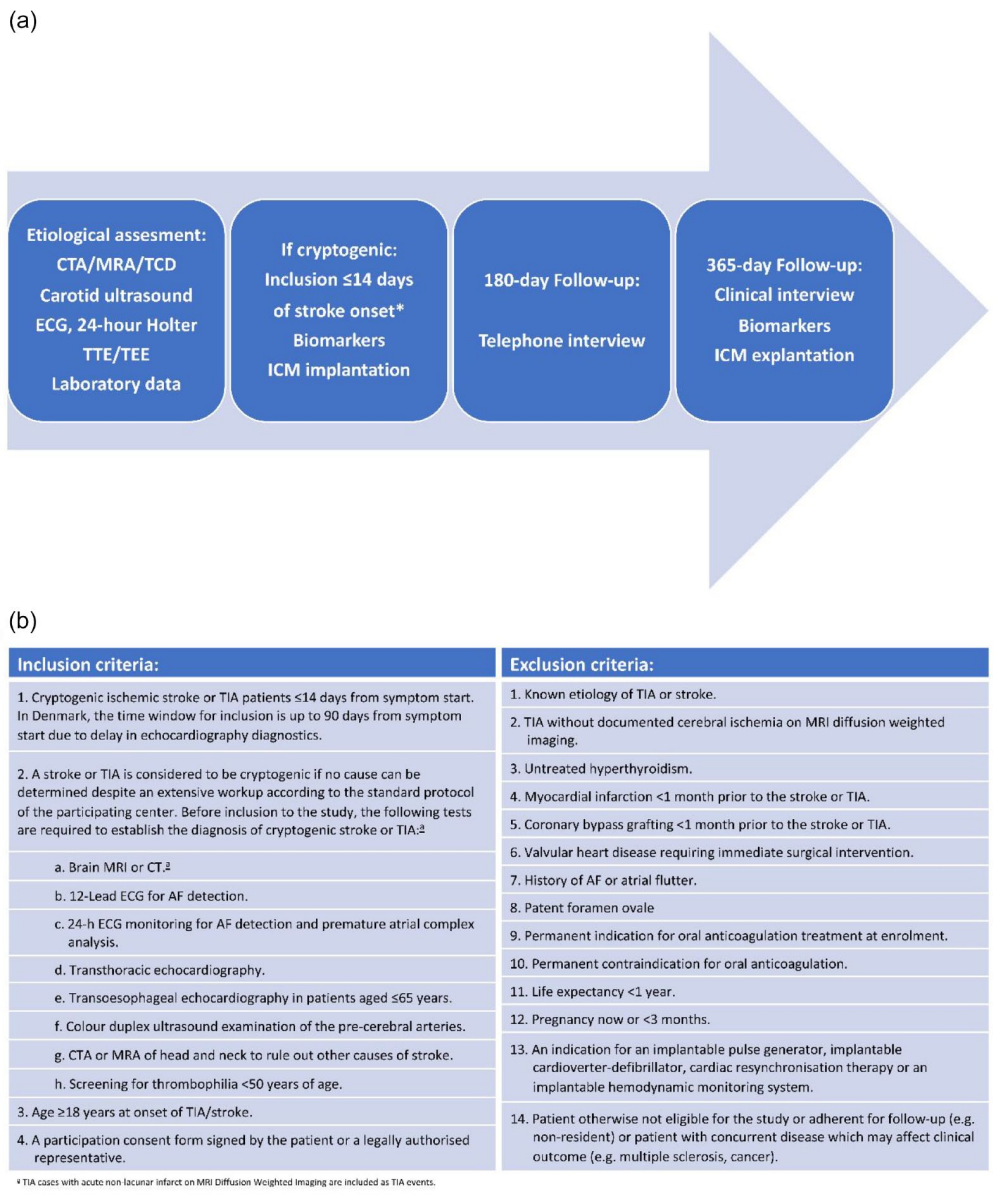
(a) Study design diagram and (b) inclusion and exclusion criteria. CTA: computed tomography angiography; MRA: magnetic resonance angiography; TCD: transcranial doppler; ECG: electrocardiography; TTE: transthoracic echocardiography; TEE: transoesophageal echocardiography; ICM: insertable cardiac monitor. *Inclusion within 3 months from stroke onset was allowed for Danish centres.^11^

The NOR-FIB Study (ClinicalTrials.gov Identifier NCT02937077, EudraCT 2018-002298-23) was a prospective, multicentre international observational real-life study collecting data from CS or cryptogenic TIA patients without previously documented history of AF.^11^ The patients were examined by a defined work-up including clinical evaluation, brain imaging, extra- and intracranial vascular evaluation, cardiac examinations, and work-up for uncommon causes before the diagnosis of CS or cryptogenic TIA was made (Figure 1). To avoid mimics, only TIA with acute lesions on diffusion-weighted magnetic resonance imaging (DW-MRI) were included.^11^ Intended time to ICM (Reveal LINQ^®^) insertion was ⩽14 days after the index event followed by remote monitoring for 12 months (Figure 1). For Danish patients the time window for inclusion was extended to 90 days due to delay in echocardiographic evaluation in this country. Periods of AF ⩾ 2 min were considered for a change of secondary prevention – a switch from antiplatelet drugs to OAC unless contraindicated. Blood samples for biomarkers were collected at enrolment and at 12-month follow-up visit.

### Study outcomes

The primary endpoint was AF detection rate within 12 months of continuous rhythm monitoring in patients with a recent CS or cryptogenic TIA without previously documented history of AF. Among the secondary endpoints were the time to first detection of AF within 6 months, CHA₂DS₂-VASc score prior to ischaemic stroke or TIA, incidence of recurrent stroke or TIA within 12 months, and use and type of OAC and antiarrhythmic drugs at the 12-month follow-up visit.

### Insertable cardiac monitors

The Reveal LINQ^®^ device used in our study is a miniaturised ICM model available in Europe since 2014, enabling rhythm classification every 2 min.^12^ The AF detection algorithm is based on an R-R interval and a P-wave evidence score. The last reduces inappropriate AF detections and is an improvement compared to predecessors. When the arrhythmia is detected the device stores 2 min of ECG, information regarding time occurrence and episode duration enabling a detailed review and quantification of AF. The LINQ is small enough for minimally invasive insertion, using the supplied insertion kit, bedside in the stroke unit or in the outpatient setting. The patients were instructed to register possible AF related symptoms like irregular heartbeats, palpitations, shortness of breath, syncope, or chest pain by using their patient activator – a small handheld device that they received at inclusion and were recommended to keep easily accessible. All investigators had access to the CareLink Network for monitoring their patients. The ECG core lab, consisting of two neurologists and two cardiologists, served to assess the quality of the monitoring recordings. ICM data of all patients were weekly reviewed and evaluated by core lab to confirm AF early. The collected information from ICM reports included quantity of AF alerts and verified AF episodes, the cumulative duration of verified AF episodes per week classified in the categories 0 (none), 1 (<2 min), 2 (2–6 minutes), 3 (>6–60 minutes), 4 (>1–24 h) and 5 (>24 h), arrhythmia symptoms and whether AF were detected during symptom alert. Once AF ⩾ 2 min was verified, local investigators were contacted to make necessary adjustment of patient’s secondary prevention (immediate OAC start, according to established treatment recommendations).

### Statistical analysis

Data were censored at the time of death; study exit or completion of 12-month follow-up. Categorical variables were presented as frequencies and corresponding percentages and continuous variables as means and standard deviations (SDs) for normally distributed variables, and medians and interquartile range (IQR) for non-normally distributed variables. Differences between groups were compared using Pearson Chi-Square test for categorical variables, and Independent Samples *t* Test or the Mann-Whitney U test for continuous variables. A *p* value <0.05 was considered significant. Statistical analyses were performed with the Statistical Package for Social Science (IBM SPSS Inc., version 26 for Windows).

## Results

Between January 2017 and September 2020, a total of 277 patients from 18 centres in Norway, Denmark and Sweden were enrolled in the study. The inclusion period was extended by 6 months due to the COVID-19 pandemic in 2020. Eighteen patients were excluded, as they did not meet the study criteria. The baseline patient characteristics are presented in Table 1. Of 259 finally assigned patients nine were lost at 12-month clinical follow-up including two patients who died (cancer and suicide) and three in whom the device was prematurely explanted. Remote monitoring ECG data from 258 and 254 patients fulfilling the 6- and 12-month rhythm monitoring periods were available for the analyses. Pre-enrolment screening for AF consisted of telemetry, Holter monitoring or R-test with a median duration of minimum 24 h (32% of patients underwent ⩾72 h ECG monitoring).

**Table 1.** Differences between AF and non-AF subgroups.

	All *N* = 259	AF group *N* = 74	Non-AF group *N* = 185	*p*-value
Age (years), mean (SD)	65.2 (±12.6)	72.6 (9.7)	62.2 (12.5)	<**0.001**
Sex (%), Female	41.7	40.5	42.2	0.811
Body mass index (kg/m^2^), no. 254, mean (SD)	26.6 (4.4)	26.0 (4.0)	26.9 (4.5)	0.128
Index event (%)				
Stroke	83.4	87.8	81.6	0.225
TIA	16.6	12.2	18.4	
Acute stroke treatment (%)				
tPA	25.9	31.1	23.8	0.226
thrombectomy	3.9	2.7	4.3	0.729
NIHSS, median (IQR)				
admission	1 (0-4)	2 (1-5)	1 (0-3)	**0.001**
discharge	1 (0-2)	1 (0-2)	0 (0-1)	**0.017**
mRS score, median (IQR)				
admission	0 (0-0)	0 (0-0)	0 (0-0)	0.382
discharge	1 (0-1)	1 (0-2)	1 (0-1)	**0.009**
CHA_2_DS_2_-VASc, median (IQR)	2 (1-4)	3 (2-4)	2 (1-3)	<**0.001**
Comorbidity:				
Hypertension* (%)	51.0	60.8	47.0	**0.045**
Diabetes mellitus* (%)	8.5	8.1	8.6	0.888
Dyslipidaemia* (%)	30.5	43.2	25.4	**0.005**
Previous stroke/TIA (%)	22.8	24.3	22.2	0.708
Heart failure (%)	1.2	1.4	1.1	1
Myocardial infarction (%)	5.8	5.4	5.9	1
Vascular disease (%)	8.5	10.8	7.6	0.398
Current smoking**(%)	22.0	10.8	26.5	**0.006**
Duration of rhythm evaluation before inclusion (%):				
24 h	52.1	52.7	51.9	0.170
48 h	15.8	9.5	18.4	
72 h	20.9	21.6	20.5	
>72 h	11.2	16.2	9.2	
Recurrent stroke or TIA at 12-month follow-up, No 250 (%)	5.6	2.7	6.8	0.363

CHA_2_DS_2_-VASc: Congestive heart failure, Hypertension, Age ⩾75 years; Diabetes mellitus, prior Stroke or TIA or thromboembolism, Vascular disease, Age 65–74 years, Sex category; HF: heart failure; TIA: transient ischaemic attack; TE: thromboembolism; MI: myocardial infarction; PAD: peripheral artery disease. mRS: modified Rankin Score. SD: standard deviation, IQR: interquartile range.

* Self-reported or use of medication at stroke or TIA onset.

** Current smoking or if stopped < 1 year ago.

### Arrhythmia detection

During the 12-month monitoring period 74 out of 259 NOR-FIB patients (28.6%) were diagnosed with paroxysmal AF or atrial flutter ⩾2 min detected by the ICM and verified by the core lab (AF alerts, tachyarrhythmia alerts, symptom alerts). The ICM was mostly inserted by a neurologist or stroke physician (91.5%). Median time from index event to insertion was 9 (IQR 7–12) days. The complication rate was low, 1.2% (pocket infection, perforation of skin, subcutaneous haematoma). Most AF (86.5%) was detected within the first 6 months from insertion: median time to the first AF episode was 24 (IQR 6–88) days, mean time 47.7 (SD 52.1) days. Arrhythmia was mainly asymptomatic: 19 of the patients with verified AF (25.7%) used the patient activator due to suspected arrhythmia symptoms as instructed, but only 6.8% of AF patients had symptom alerts verified as AF. Totally 47.3% of AF patients, when asked at 12-month follow-up, mentioned to have experienced some suspected symptoms. However, only half of these patients used their patient activators. One in seven patients mentioning symptoms had a verified AF in their symptom alerts.

- A total amount of 17 478 AF alerts in 112 patients was detected. Of those, 4927 alerts (28.2%) in 68 patients were verified as AF or atrial flutter episodes. The shortest AF was 2 min and the longest >99 h.
- During the monitoring period, 71 patients (27.4%) used their patient activators, in whom 1289 symptom alerts were registered. Most symptom alerts showed no significant arrhythmia (sinus tachycardia, supraventricular tachycardia, artefacts). AF ⩾ 2 min were seen in five of those 71 patients (7%).
- AF was detected in 35.1% of the AF patients also during tachyarrhythmia alerts, in 8.1% in tachyarrhythmia episodes only (no additional AF alerts).

**Figure 2.**
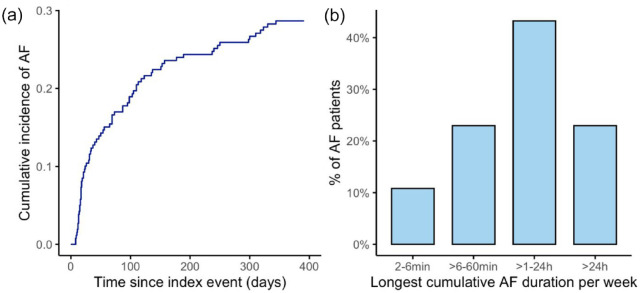
Incidence and burden of detected AF in the NOR-FIB study: (a) cumulative incidence of AF within 12 months monitoring; (b) longest cumulative AF burden per week during follow-up.

Detected arrhythmia was recurrent in 91.9%. Median AF episodes count was 17 (IQR 4–66), mean 71 (SD 142). Median count of weeks with AF arrhythmia was 8 (IQR 3–25) while mean count of weeks with AF was 15 (SD 14.7). In two out of three patients longest cumulative AF duration per week was ⩾1 h (Figure 2), while 63.5% of all AF patients had episodes lasting ⩾1 h per day.

OAC was recommended to all patients with verified AF or atrial flutter. At 12-month follow-up 72 of 74 patients (97.3%) were on OAC therapy. The factor Xa inhibitors were used in most cases (83.3%). There was no difference in the use of antiarrhythmic drugs in the AF and non-AF groups, 20.5% versus 11.3% (*p* = 0.149). Selective beta blockers were used most often. Patients in the AF group had higher premorbid CHA_2_DS_2_-VASc score (*p* < 0.001), were older (*p* < 0.001), and had more often hypertension (*p* = 0.045) and dyslipidaemia (*p* = 0.005). NIHSS scores on admission and discharge in AF patients were higher compared to non-AF patients (*p* = 0.001 and *p* = 0.017). More details are presented in Table 1. During the follow-up period of 12 months two strokes occurred in the AF group (both before first AF episode was detected) and twelve in the non-AF group, however the difference was not statistically significant.

## Discussion

In the present study, we found that ICM administered by the stroke units was an effective tool for detection of underlying AF, capturing the arrhythmia in 29% of CS and cryptogenic TIA patients. The novel feature of the NOR-FIB study is that insertion and use of ICM was tested fully practicable for stroke physicians, with clinical implication that this approach may easily be implemented in stroke unit evaluation of CS.

**Figure 3.**
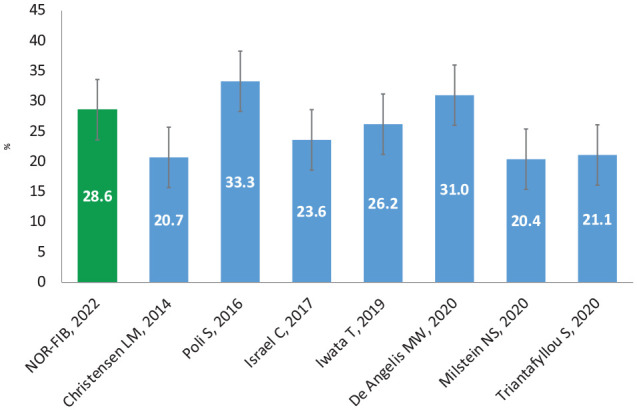
Twelve-month AF detection rate with 95% confidence intervals, studies on CS patients using ICM, AF duration ⩾2 min.

The AF rate findings of our study are consistent with results from similar CS studies, published after our study was intended, using the same device (Figure 3).^7,13–18^

However, the AF detection rate at 12-month follow-up in the NOR-FIB was considerably higher than in the earliest studies using ICM for cardiac rhythm monitoring after CS. In the CRYSTAL AF study, the AF detection rate was 12.4% whereas in the SURPRISE study (569 (±310) days of follow-up) 20.7%.^6,7^ This may be explained by improved selection criteria using extensive pre-enrolment evaluation and improved detection algorithm in the newest devices.^11,12^ The high AF rate is also consistent with findings from the last meta-analysis of 23 studies including 3 472 CS patients^9^ where the overall proportion of AF detected by ICM was 25%, suggesting that evaluation approach used in the NOR-FIB study was good. As common high-risk factors and mechanisms of stroke can be systematically excluded (i.e. carotid stenosis, PFO) before the diagnosis CS is made and ICM is inserted,^11,19^ this evaluation strategy is in our opinion robust enough for the current practice. With completed work-up before ICM insertion and early start of monitoring for 1 year, the rate of missing occult AF in CS patients is supposed to be low.

There is currently no universally accepted burden of AF in the population after stroke. Furthermore, the apparent benefit of anticoagulation initiation in patients with AF detected by ICM is still debated due to the lack of studies showing an improved outcome.^20^ However, OAC is recommended for patients with stroke or TIA regardless of the amount of time spent in AF.^5^ The definition of AF episodes ⩾2 min in our study was based on investigator consensus and technical consideration, and neither it nor the more liberal definition with arrhythmia duration of ⩾30 s have been validated. We focused on finding AF episodes ⩾2 min, as there were previously raised doubts about subclinical AF of shorter duration and its significance for thromboembolic events. However, the same consideration usually does not include stroke patients diagnosed with AF (often asymptomatic and of short duration too) in hospital or on Holter monitoring. Those patients are usually being anticoagulated regardless of considering future AF episodes, as the monitoring is cessed as soon as the arrhythmia is found. Answering the next question, whether detected AF is the bystander, cause, or consequence of the brain impact on the heart in the acute phase, our study demonstrates a recurrent nature of the arrhythmia in 91.9% of the cases and the longest weekly AF burden of hours to days in two-thirds of the patients (Figure 2). In previous studies, episodes of such duration were associated with 3 to 9 fold increases in stroke risk.^20^ The NOR-FIB patients with AF detected early after stroke also had episodes sustained over several months. These findings are strengthening the suspicion of subclinical AF episodes detected by ICM to be causally related to stroke and contributing to a better recognition of the significance of cardiac arrhythmias in CS. Causally related is not necessary the same as stroke timely related to AF, which is known from previous studies and hypothesis that thromboembolism in AF is not only reflected by the length or frequency of arrhythmia but also by atrial myopathy, AF being a marker of,^21^ and co-morbidities as shown in the meta-analysis.^22^

We have, however, limited the follow-up period to 12 months as there are uncertainties about the significance of AF episodes detected long after the index stroke. For timeframe longer than 12 months in cases where AF is not found, further observation most likely represents screening for AF in high-risk patients rather than for AF as the underlying stroke etiology, though final treatment is the same and AF detection is therefore still important.^20,23^

Approximately 70% of all AF alerts were false positive. Two neurologists responsible for ECG evaluation filtered all AF, tachyarrhythmia, symptom, or other registered arrhythmia alerts for further evaluation by cardiologists. This led to reduced workload for cardiologists that could focus just on selected cases and not on patients without episodes or those with detected arrhythmias caused by mainly under sensing. Unclear episodes were always evaluated by two cardiologists and AF diagnosis was always made by cardiologist. In addition, to secure high quality of this study, after 12 months ECG registration was completed, all episodes were again reviewed by core lab before final analyses of the NOR-FIB data were performed. Pre-screening of episodes together and evaluation with cardiologists varied throughout the study due to changing numbers of observed patients, patient to patient variability in terms of number of episodes and could vary between 2 and 5 h per week.

Our finding of a high AF detection rate underscores the need for intensification of the cardiac rhythm monitoring for CS and cryptogenic TIA patients. The recently presented *European Stroke Organisation guideline on screening for subclinical atrial fibrillation after stroke or transient ischaemic attack of undetermined origin* recommends longer duration of cardiac rhythm monitoring of more than 48 h with ICM if feasible to increase the detection of subclinical AF.^24^ This recommendation, long awaited among stroke physicians, is an important step in stroke prevention for a long-neglected patient group. The guideline emphasises the importance of early initiation of monitoring in eligible CS and cryptogenic TIA patients as the best way to prevent stroke. To secure proper access to and avoid delay in planned investigations performed by other specialists a better interdisciplinary and intersectoral coordination is needed. With the stroke physicians, taking a more active role and performing the ICM insertion bedside in the stroke unit, early start of advanced cardiac rhythm diagnostic can be accessible for most eligible patients, as shown in our study. The high clinical relevance is that implementation of ICM usage in stroke units would lead to much faster monitoring than patient referral to cardiologic clinic. Weekly monitoring of incoming ECG reports once ICM is inserted, as performed in our study, seems reasonable for early confirmation of the AF diagnosis, and change of secondary prevention. Daily evaluation would have been even better but may somehow be difficult to comply in a clinical setting, while weekly monitoring seems more realistic. Upcoming artificial intelligence (AI) based solutions may significantly reduce the time and effort needed to adjudicate false-positive events.

### Limitations

One of the limitations is the time of patient recruitment for the Danish centres: ICM insertion allowed <3 months after index event, due to delay in cardiac diagnostic as previously explained.^11^ Median time from stroke onset to ICM insertion for Danish patients was 42 (IQR 27–70) days, considerably higher than for the whole NOR-FIB group (9 (IQR 7–12) days). However, Danish patients represent only 6.2% of our population thus the prolonged inclusion does not weaken our results substantially. Some of those patients may have had AF detected earlier by shortening this time window. Regular use of ICMs in stroke evaluation should thus aim for early start of monitoring as soon as the diagnosis of CS is made. Although one-third of the patients had ⩾72 h ECG monitoring before ICM insertion, the minimum duration of pre-enrolment cardiac rhythm monitoring was of 24 h according to the current guidelines at the start of the study. It could be argued that increasing the length of the monitoring for more than 48 h could possibly reveal AF in some included patients before ICM insertion and would have been more cost-effective. However, only three patients had their AF detected within 2 days after ICM insertion and five patients within 14 days after index stroke.

The rate of symptomatic AF may have been higher. As mentioned earlier, the true AF rate in symptomatic episodes among patients with symptoms as well as AF patients was low. Consequently, we only chose patients that used the patient activator to evaluate the rate of symptomatic episodes, since it is difficult to objectively relate the subjective symptoms to detected AF episodes.

The sample size of the study and follow-up time may not show the real difference for stroke risk recurrence in favour of OAC treated AF patients vs patients in whom AF was not detected. This may be even true for the 27.8% of NOR-FIB patients not having any arrhythmia alerts registered during the whole monitoring period and probably at lower risk of cardioembolic stroke. On the other hand, the patients were followed to detect also other causes than AF and optimised secondary prevention may also have lowered recurrence risk in the non-AF group. The study was not designed to demonstrate reduction in stroke recurrence risk using ICM. The evidence supporting that ICM usage is associated with lower stroke recurrence risk is still lacking.^10^

At last, patient inclusion was up to the discretion of local investigators according to the inclusion and exclusion criteria. Study protocol stated that only stroke patients without etiology after protocolled work-up should have been included in the study.^11^ Lacunar strokes were not included as the etiology is mainly microangiopathy. However, lacunar strokes may also occur in patients with atrial fibrillation and small cardiac embolies.

## Conclusion

Paroxysmal AF was considered the most probable cause in a substantial part of the CS and TIA patients in the NOR-FIB study. Prolonged cardiac rhythm monitoring with ICMs was an effective tool for diagnosing an underlying AF and is feasible to be implemented in stroke unit evaluation of cryptogenic ischaemic events. Following the recent ESO guideline and the implementation of ICM in the stroke units, this can potentially lead to decreased CS rates if larger proportions of paroxysmal AF patients are detected. Accordingly, stroke recurrence can hopefully be reduced if more patients with paroxysmal AF are treated with anticoagulants. However, RCTs demonstrating reduced stroke rates are still outstanding.
